# Loss of *Drosophila* Vps16A enhances autophagosome formation through reduced Tor activity

**DOI:** 10.1080/15548627.2015.1059559

**Published:** 2015-09-14

**Authors:** Szabolcs Takáts, Ágnes Varga, Karolina Pircs, Gábor Juhász

**Affiliations:** 1 Department of Anatomy; Cell and Developmental Biology; Eötvös Lorand University; Budapest, Hungary; 2 Momentum Drosophila Autophagy Research Group; Institute of Genetics; Biological Research Center; Hungarian Academy of Sciences; Szeged, Hungary

**Keywords:** autophagy, flux, HOPS, lysosome, Syntaxin 17, Tor, Vps16A

## Abstract

The HOPS tethering complex facilitates autophagosome-lysosome fusion by binding to Syx17 (Syntaxin 17), the autophagosomal SNARE. Here we show that loss of the core HOPS complex subunit Vps16A enhances autophagosome formation and slows down *Drosophila* development. Mechanistically, Tor kinase is less active in *Vps16A* mutants likely due to impaired endocytic and biosynthetic transport to the lysosome, a site of its activation. Tor reactivation by overexpression of Rheb suppresses autophagosome formation and restores growth and developmental timing in these animals. Thus, Vps16A reduces autophagosome numbers both by indirectly restricting their formation rate and by directly promoting their clearance. In contrast, the loss of Syx17 blocks autophagic flux without affecting the induction step in *Drosophila*.

## Abbreviations

EIF4EBP1: eukaryotic initiation factor 4E-binding protein
Atg: autophagy related
car: carnation
Cp1: cysteine proteinase-1
CORVET: class C core vacuole and endosome tethering
C-Vps: class C Vps
DAPI: 4′,6-diamidino-2-phenylindole
Gal4: galactose metabolism 4
dor: deep orange
gig/Tsc2: gigas/tuberous sclerosis complex 2
HOPS: homotypic fusion and protein sorting
IGF: insulin-like growth factor
lt: light
PtdIns3K: class III phosphatidylinositol 3-kinase
ref(2)P: refractory to sigma P
Rheb: Ras homolog enriched in brain
S6k: S6 kinase
SNARE: soluble NSF attachment protein receptor
Syx17: Syntaxin 17
Tor: target of rapamycin
TFEB: transcription factor EB
UAS: upstream activation sequence
Vps: vacuolar protein sorting.

## Introduction

In all eukaryotic cells, Tor (target of rapamycin) kinase is a central regulator of cell growth and autophagy, a catabolic process responsible for lysosomal degradation and recycling of bulk cytosol and organelles.^1^ In the presence of adequate nutrients and growth factors, Tor complex 1 promotes translation and cell growth at least in part by phosphorylating and activating S6k/RPS6KB1 and inactivating Thor/EIF4EBP1 (eukaryotic initiation factor 4E-binding protein; a suppressor of translation initiation), and downregulates autophagy by inhibitory phosphorylation of Atg1 homologs, which are upstream factors required for autophagosome biogenesis. Starvation or growth factor withdrawal inhibits Tor, and as a result it blocks cell growth and activates Atg1, which then promotes autophagosome formation by phosphorylating other Atg proteins such as Atg13 in *Drosophila*.^2,3^ It has been established that the activation of Tor takes place on lysosomes, and that Tor is recruited to these organelles through binding to the lysosome-anchored Ragulator complex.^4,5^ Its activity also depends on sensing the presence of amino acids inside the lysosome, in association with the vacuolar H^+^-ATPase.^6^ In line with this model, Tor is reactivated during prolonged starvation in an autophagy-dependent manner, presumably as a result of restoration of amino acid levels within lysosomes by autophagic breakdown of proteins.^7^

Others and we have recently shown that the fusion of autophagosomes with lysosomes is mediated by a Syx17 (Syntaxin 17)-containing SNARE complex, both in mammals and *Drosophila*.^8,9^ Interaction of the HOPS (homotypic fusion and protein sorting) tethering complex with the autophagosomal SNARE Syx17 promotes the tethering of autophagosomes with lysosomes in animal cells.^10,11^ Thus, the HOPS-Syx17 axis is required for lysosomal degradation of autophagic cargo, which is often referred to as autophagic flux. We also found that *Drosophila* Syx17 is largely dispensable for the fusion of endosomes with lysosomes and for biosynthetic transport to lysosomes, unlike HOPS.^11^

In this work, we present the further analysis of the essential HOPS subunit Vps16A. We find that autophagosome formation is accelerated in *Vps16A*-mutant *Drosophila* larvae, likely as a result of defective endo-lysosomal trafficking leading to impaired Tor activation. Therefore, loss of Vps16A increases autophagosome numbers not only due to a block of their clearance, but also by promoting their rate of formation.

## Results

**Figure 1.**
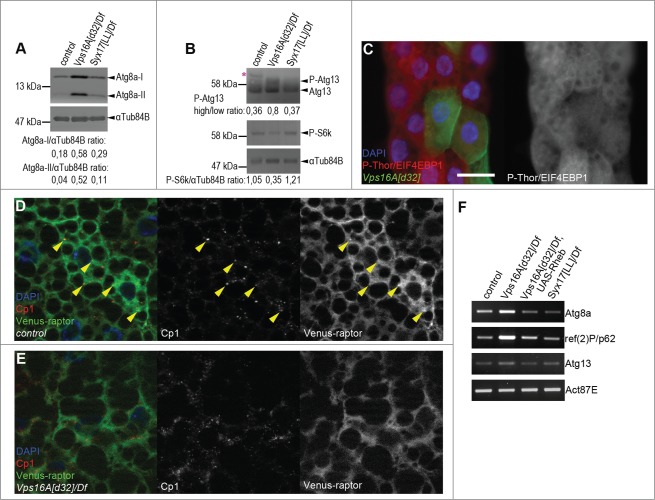
Reduced Tor activity in *Vps16A* mutants. (**A**) Representative western blots show that the levels of lipidated, autophagosome-associated Atg8a-II are much higher in well-fed *Vps16A* mutants than in control or *Syx17*-mutant L3-stage larvae. (**B**) The levels of P-S6k/Phospho-RPS6KB1 (Thr389) are reduced, while the ratio of hyperphosphorylated (slower migrating) to hypophosphorylated (faster migrating) Atg13 is increased in *Vps16A* mutants, compared to well-fed control or *Syx17*-mutant larvae. Asterisk marks a nonspecific band. (**C**) The level of P-Thor/Phospho-EIF4EBP1 (Thr37/46) is decreased in GFP-marked *Vps16A*-null mutant cells compared to neighboring control cells in mosaic fat bodies of well-fed larvae. (**D**) Venus-tagged raptor displays punctate localization in fat body cells of well-fed wild-type larvae, and colocalizes with Cp1 (Cathepsin L)-positive lysosomes (arrowheads). (**E**) Punctate localization of Venus-raptor is lost in *Vps16A* mutants. (**F**) RT-PCR analysis reveals the obvious transcriptional upregulation of *Atg8a* and *ref(2)P/p62* in *Vps16A* mutants when compared to control or *Syx17*-mutant animals. Overexpression of Rheb in *Vps16A* mutants restores *Atg8a* and *ref(2)P/p62* mRNA levels to those seen in control animals. Scale bar equals 20 µm for panels (**C-E**). Numbers represent protein level ratios estimated by densitometry in panels (**A** and **B**).

We and others have recently established that autophagic flux is impaired upon loss of Syx17 or HOPS function, both under basal and starvation conditions.^8-12^ Interestingly, western blots of well-fed *Drosophila* larvae revealed much higher levels of autophagosome-associated, lipidated Atg8a-II in *Vps16A* mutants than in *Syx17*-mutant larvae (**Fig. 1A**), even though autophagosome-lysosome fusion is blocked in both cases. As the activation of Tor takes place on lysosomes, we hypothesized that impaired endocytic and biosynthetic transport to lysosomes may be responsible for this phenotype in *Vps16A* mutants. In line with this, the level of P-S6k/Phospho-RPS6KB1 (Thr389) was reduced and the amount of hyperphosphorylated Atg13 was increased in *Vps16A* mutants (**Fig. 1B**). Moreover, the level of P-Thor/Phospho-EIF4EBP1 (Thr37/46) was cell-autonomously decreased in *Vps16A*-null mutant fat cells in mosaic animals (**Fig. 1C**). These data altogether indicate reduced Tor and increased Atg1 kinase activity in *Vps16A*-mutant cells, respectively.

Next we sought to determine the localization of Tor in *Vps16A* loss-of-function cells. There are 2 different MTOR kinase complexes in eukaryotic cells, MTOR complex 1 and 2. These 2 complexes can be distinguished based on the presence of RPTOR/raptor (complex 1) and RICTOR (complex 2).^1^ The RPTOR-containing complex 1 is involved in the regulation of cell growth and autophagy, so we generated transgenic flies expressing Venus-tagged raptor to be able to follow its subcellular localization. Raptor displayed punctate distribution in fat body cells of well-fed control animals, and these dots colocalized with Cp1 (cathepsin L)-positive lysosomes (**Fig. 1D**). In contrast, the punctate localization of raptor was largely lost in *Vps16A* mutants (**Fig. 1E**), further supporting our model of impaired Tor activation.

Interestingly, *Vps16A* mutants had increased levels of not only the lipidated, autophagosome-associated Atg8a species, but also higher amounts of the nonlipidated form when compared to wild-type or *Syx17*-mutant tissues (**Fig. 1A**), suggesting that the transcription rate of this gene may also increase. Moreover, the protein level of the selective autophagy cargo ref(2)P/SQSTM1/p62 also increased, and these changes appeared to be specific, as we could not detect an upregulation of Atg1 or Atg9, which are involved in the early steps of autophagosome formation (**Fig. S1**). RT-PCR experiments revealed a remarkable increase in the mRNA levels of both *Atg8a* and *ref(2)P*, unlike in the case of *Atg13* (**Fig. 1F**), which is in line with our western blot data.

**Figure 2.**
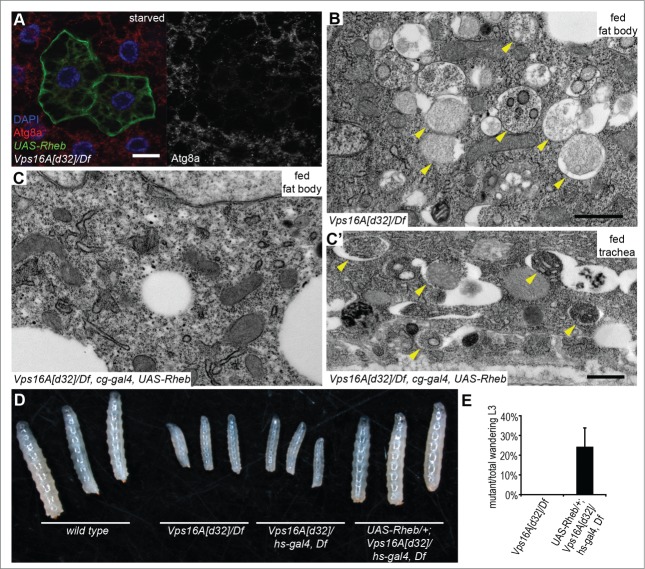
Reactivation of Tor suppresses autophagosome formation and restores growth and developmental timing in *Vps16A* mutants. (**A**) Overexpression of Rheb in GFP-positive cell clones suppresses endogenous Atg8a-positive autophagosome formation in starved *Vps16A*-mutant fat body cells. (**B**) Ultrastructural analysis shows the large-scale accumulation of double-membrane autophagosomes (arrowheads) in fat body cells of well-fed *Vps16A*-mutant larvae. (**C and C'**) Fat body-specific expression of Rheb driven by *cg-Gal4* suppresses autophagosome formation in fat cells (**C**) but not in the tracheal epithel (**C'**) of well-fed *Vps16A*-mutant larvae. (**D**) The size defect of *Vps16A*-mutant larvae 4 d after egg laying is rescued by low-level expression of Rheb. (**E**) Low-level expression of Rheb using an uninduced *hs-Gal4* driver rescues developmental timing, as assessed by the ratio of wandering L3-stage larvae 5 d after egg laying, compared to heterozygous siblings. N=15 and N=12 culture vials were evaluated to calculate data for *Vps16A* mutants and Rheb-expressing *Vps16A* mutants, respectively, with an average of 30 larvae counted per vial. Note that the expected Mendelian ratio is 33%. Scale bars :20 µm (**A**), and 1 µm (**B, C, C'**).

We hypothesized that restoring Tor activity may suppress the enhanced autophagosome formation that we observed in *Vps16A* mutants. Tor is regulated by small GTPases including Rheb. Overexpression of Rheb has been shown to inhibit autophagy through activation of Tor in *Drosophila* fat cells.^13^ Indeed, Rheb overexpression led to a near-complete elimination of autophagosomes based on endogenous Atg8a puncta formation in *Vps16A* mutants even after 4 h of starvation (**Fig. 2A**). The result of this genetic epistasis test was confirmed on the ultrastructural level: fat body-specific overexpression of Rheb in *Vps16A*-mutant animals suppressed the accumulation of autophagosomes, both in well-fed and 4 h-starved larvae (**Fig. 2B, C**; **Fig. S2A, B**). As expected, this effect of Rheb expression was specific for fat body cells, as autophagosome accumulation was not suppressed in other tissues of *Vps16A*-mutant larvae such as the tracheal epithelium (**Fig. 2C′**; **Fig. S2B′**). Overexpression of Rheb strongly promotes cell growth in diploid cells such as the ones found in imaginal disc epithelium, but its growth effect is not seen in the fat body, likely because these polyploid cells grow 200-fold during the 4 d of larval development and their Tor activity may already be close to maximal. To analyze this aspect of the genetic relationship between *Vps16A* and *Rheb*, we took advantage of our previous observations that that the growth-promoting effect of Rheb becomes obvious even in the fat body if larvae are starved for 48 h in water, similar to the phenotypes produced by increased insulin signaling in mosaic animals.^14,15^ Indeed, either the overexpression of Rheb or RNAi-silencing of its inhibitor *gig/Tsc2* were able to enhance the growth of fat cells in 48 h-starved *Vps16A*-mutant larvae as well as in control animals (**Fig. S2C-F**). Rheb expression also restored the levels of *Atg8a* and *ref(2)P* mRNA in *Vps16A* mutants to that seen in wild-type animals (**Fig. 1F**), indicating that it is reduced Tor activity in the mutants that leads to enhanced transcription of these genes.

Importantly, low-level expression of Rheb also rescued the growth defect observed in *Vps16A*-mutant larvae (**Fig. 2D**). Wild-type *Drosophila* larvae initiate wandering behavior on d 5 after egg laying, when animals leave the food in search of a dry place to pupariate. Whereas none of the *Vps16A*-mutant larvae were wandering at this time, Rheb expression rescued this developmental delay as well (**Fig. 2E**). Rheb expression did not rescue the fully penetrant late larval lethality of *Vps16A* mutants, though, which is likely due to a block of multiple trafficking routes to lysosomes.

Reduced levels of the molting hormone ecdysone and increased IGF (insulin-like growth factor) and class III phosphatidylinositol 3-kinase (PtdIns3K) signaling were reported in fat body cells of wandering stage, hypomorphic, hemizygous *dor* (*deep orange*) mutant larvae.^16^ As this gene encodes the fly homolog of another HOPS-complex subunit (dor/Vps18), the potential role of similar alterations was tested in *Vps16A* mutants. We found no change in IGF and PtdIns3K signaling in well-fed or wandering stage *Vps16A* mutants compared to controls using the same reporter for detecting the activation of PtdIns3K (data not shown). Moreover, ecdysone treatment did not restore developmental timing and the initiation of wandering behavior in *Vps16A* mutants (data not shown), suggesting that the observed developmental delay is not caused by reduced ecdysone or increased IGF and PtdIns3K signaling.

Lt/Vps41 homologs are thought to be HOPS-specific subunits in animal cells, as another evolutionarily conserved protein (CG10144/Vps8) may play its role as part of a related complex called CORVET (class C core vacuole and endosome tethering).^17,18^ CORVET promotes the maturation of early to late endosomes in yeast but remains poorly characterized in animals. Metazoan HOPS and CORVET tethering complexes are speculated to share 5 subunits: the homologs of CG32350/Vps11, Vps16, dor/Vps18, car/Vps33, and Vps39.^17,18^ We have recently shown that autophagosomes accumulate when any one of the 6 HOPS subunits are lost in *Drosophila*, and the levels of both lipidated and nonlipidated forms of Atg8a and ref(2)P/p62 are massively upregulated not only in *Vps16A*-null mutants but also in hypomorphic *CG32350/Vps11*- or *lt/Vps41*-mutant animals.^11,19^ Importantly, *CG32350/Vps11*-mutant larvae also developed slower than control animals, which could be again partially rescued by the expression of Rheb (**Fig. S2G**), implying that it is caused by a similar mechanism as in the case of *Vps16A* mutants. Moreover, *lt*-mutant larvae developed slower than their heterozygous siblings, too (**Fig. S2H**). All these similar phenotypes suggest that the effects of *Vps16A* mutations on Tor and autophagy may be due to impaired HOPS function.

Collectively, our results indicate that reduced Tor activity is responsible for the enhanced autophagosome formation and delayed larval development of *Vps16A* mutants.

## Discussion

Drugs inhibiting lysosomal degradation, such as chloroquine, are routinely used in autophagic flux experiments. In these tests, the degradation of a specific autophagic cargo (for example the lipidated, autophagosome-associated form of Atg8 family proteins) is estimated from the increase of its levels in the presence of such a drug, compared to the condition without drug treatment.^20^ However, these lysosome inhibitors were proposed to increase the rate of autophagosome formation by lowering MTOR activity.^21^ Recent papers provided experimental support for this theory.^22,23^ Thus, the loss of certain genes involved in endocytic or biosynthetic transport to lysosomes may cause similar effects as these lysosome inhibitors. Our results showing that the essential HOPS subunit Vps16A is required for full Tor activity and suppression of autophagosome formation are perfectly in line with this model.

This mechanism of Tor activation is also supported by recent genetic experiments in yeast. The 2 related endomembrane tethering complexes, HOPS (involved in trafficking to the lysosome and vacuole), and CORVET (mediating early to late endosome transport) share a common core termed class C Vps (C-Vps), which consists of CG32350/Vps11, ird1/Vps15, dor/Vps18, and car/Vps33.^17,18^ Two additional subunits specify HOPS (Vps39, lt/Vps41) and CORVET (Vps3, CG10144/Vps8) in this unicellular eukaryote, respectively, although intermediate complexes may also exist. Mutations in genes encoding C-Vps subunits are synthetic lethal with *tor1* mutations (note that there are 2 different genes encoding Tor in yeast, and it is Tor1 that is part of Tor complex 1), and C-Vps mutants are hypersensitive to rapamycin, suggesting a role for this complex in Tor complex 1 signaling.^24^ It has recently been established that Tor1 activity is decreased to about 30% in C-Vps-mutant yeast cells compared to controls,^25^ which exactly matches the reduction of P-S6k levels seen in *Vps16A*-mutant *Drosophila* (shown in **Fig. 1B**). The loss of HOPS-specific subunits has a similarly strong effect on Tor1 activity as it is reduced to about 45%, whereas the loss of CORVET-specific CG10144/Vps8 only slightly affects Tor1 (about 80% of its activity remains). Genetically promoting Tor1 signaling restores its activity and rapamycin recovery in C-Vps mutants, which also indicates that this complex functions upstream of Tor1 in yeast.^25^ Importantly, yeast cells lacking HOPS subunits grow normally in rich medium despite reduced Tor1 activity, and their growth defect only becomes apparent when Tor1 is mutated or inhibited by rapamycin, which may be in part due to the block of prosurvival autophagic degradation.^25^ These data are in line with our observations that Tor complex 1 activity is cell-autonomously reduced in *Vps16A*-mutant cells, but this does not significantly affect their growth. It is important to emphasize that the small number of mutant cells in mosaic *Drosophila* larvae have access to nutrients and growth factors from the hemolymph, and may be able to grow normally in this rich environment despite their reduced Tor activity and increased autophagosome formation. In addition, the net growth of cells is determined by the opposing effects of 2 processes: the generation of new biomass versus shrinkage due to the catabolism of self-material. In HOPS-mutant cells autophagosomes form but are not cleared, so this major route of cell shrinkage is blocked. Of note, the delayed growth phenotypes of *Drosophila* HOPS mutants become obvious on the organismal level, which can even be partially rescued by increasing Tor activity through expression of Rheb, despite the impaired trafficking and degradation of autophagic, endocytic, and biosynthetic transport to lysosomes in all cells. This again indicates that similar mechanisms may be involved in regulating Tor/MTOR activity in yeast and animal cells.

Interestingly, the overall levels of both Atg8a and ref(2)P/SQSTM1/p62 proteins raise massively in HOPS mutants relative to not only control but also to *Syx17*- or *Atg7*-mutant larvae, based on this study and our recent publications.^11,19^ The data presented here suggest that this is in part due to the enhanced transcription of these 2 genes, which is again suppressed by the expression of Rheb. While the mechanism responsible for such changes is not known, it may involve the activation of the *Drosophila* homolog of TFEB, as this transcription factor is known to upregulate several genes involved in autophagy and lysosomal activity in mammalian cells.^26^ TFEB is phosphorylated by MTOR and is also associated with the lysosomal surface in well-fed cells, and it gets released from there when MTOR is inactivated and released, enabling TFEB to translocate to the nucleus and induce the expression of its target genes.^27-30^ Moreover, the transcription factor foxo/FOXO is a major positive regulator of autophagy both in fruit flies and mammals, and it was implicated in the transcriptional upregulation of numerous *Atg* genes during the starvation response in *Drosophila* larvae.^31,32^ Of note, *Atg8a* and *ref(2)P/SQSTM1/p62* are also among the most highly induced genes under starvation,^32^ probably because the corresponding proteins are selectively degraded by autophagy and hence there is a need for their enhanced synthesis.

**Figure 3.**
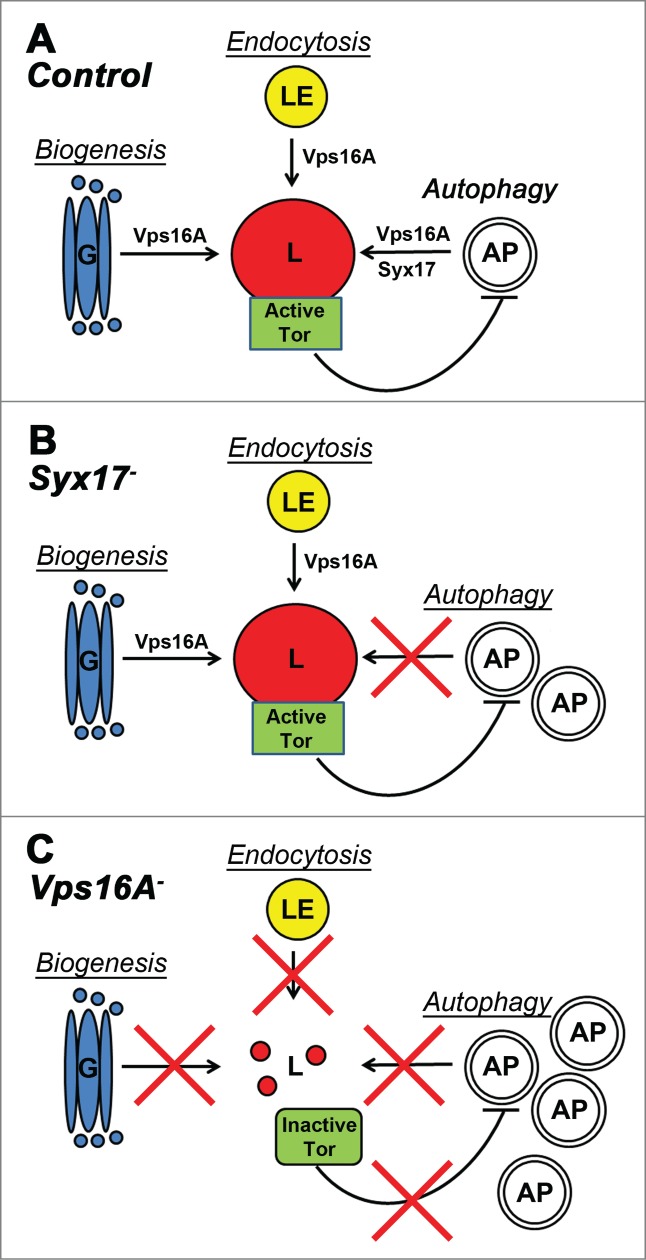
A model of Vps16A function. (**A**) Vps16A is required for multiple transport routes to lysosomes, including their biogenesis, endocytosis, and autophagy. (**B**) Loss of Syx17 blocks autophagosome-lysosome fusion and autophagic flux without affecting lysosome-dependent Tor activation. (**C**) Loss of Vps16A inhibits all 3 transport routes to lysosomes, which results in decreased Tor activity and increased autophagosome formation, in addition to a block of autophagosome-lysosome fusion. AP, autophagosome; G, Golgi; L, lysosome; LE, late endosome.

In summary, in addition to the well-established role for Vps16A and HOPS downstream of Tor and autophagy signaling in autophagosome clearance, we showed that Vps16A also functions upstream of Tor in *Drosophila*, and it suppresses autophagosome formation by ensuring maximal Tor activity. Thus, Vps16A reduces autophagosome numbers both by indirectly inhibiting their rate of formation and by directly promoting their fusion with lysosomes. While *Vps16A*-mutant phenotypes are similar to the effects of drugs inhibiting lysosomal degradation in many ways, the loss of Syx17 appears to block autophagic flux without affecting the induction step (**Fig. 3**). These data suggest that in *Drosophila, Syx17* mutation or RNAi may turn out to be an even better tool for autophagic flux experiments than drug treatment to inhibit lysosomal degradation.^12^

## Materials and Methods

### *Drosophila* genetics

Flies were reared on standard cornmeal-agar-yeast medium. Mutant and overexpression lines used in this study were *UAS-Rheb, hs-Gal4, Df(3R)BSC507, Df(3R)ED5339, Df(3L)Exel8098, Df(2L)lt45* (all obtained from the Bloomington Stock Center*), UAS-gig/Tsc2 RNAi[KK100646]* (obtained from the Vienna *Drosophila* RNAi Center)*, CG32350/Vps11[LL06553], lt[11], Vps16A[d32]*,^11^ *Syx17[LL06330]*,^9^ *hs-Flp, Act>CD2>Gal4, UAS-mCD8-GFP* (all recombined together on chromosome X),^3^ and *UAS-Venus-raptor* (this study). Expression of Venus-raptor was mediated by crossing transgenic flies to a *hs-Gal4* stock, and heat shocking early L3-stage larvae for 30′ at 37°C, followed by a 4 h recovery. *Vps16A* null mutant clones were generated using the mosaic analysis with a repressible marker (MARCM) method, by crossing *FRT82B Vps16A[d32]* mutants with *hs-Flp; QUAS-mCD8-GFP; ET49-QF, FRT82B tub-QS* flies and heat shocking 0 to 4 h embryos in a 37°C water bath for 1 h, as before.^3,11^

### Molecular cloning and PCR

*Raptor* coding sequences were PCR amplified from genomic DNA using primers ATT ACA AGT GCC GTT CGC GCT AAG T and ATC TTA AGA TTG TTC TCT ACC TGG GGT GGG and blunt cloned into XmnI-EcoRV digested, dephosphorylated pENTR1A (Invitrogen, 11813-011), followed by recombination into pTVW (DGRC, 1091). Transgenic flies were established by standard embryo transformation. For reverse transcriptase-PCR, we isolated total RNA using PureLink RNA Mini Kit (Invitrogen, 12183018A) from L3-stage larvae 3 h after a 1-h heat shock to induce Rheb expression in *Vps16A* mutants (all genotypes were treated equally and received a heat shock). This was followed by cDNA synthesis with RevertAid First Strand cDNA Synthesis Kit (Fermentas, K1622) and PCR amplification for 25 cycles. These primers were used in the reactions: CAGCGAAAGTGTTGATTTGG and AGGGCGTAACCCTCGTAGAT (*Act87E*), TGGATCGACGCTGATAAAGA and GTCTCCTGAAACGGGCAAT (*ref(2)P*/*SQSTM1*/*p62*), GTCGCAAATATCCAGACCGT and GCCCTGCGTATCAGATCAAT (*Atg8a*), GCGTGGAAATATCCCTGAAA and TGGTGTTGCTCTCTGACTGG (*Atg13*).

### Histology and western blots

Transmission electron microscopy was carried out as before.^9,11^ Indirect immunofluorescent labeling and microscopy was carried out as described previously, using chicken anti-GFP (Invitrogen, A10262), rabbit anti-Cp1/cathepsin L (Abcam, ab58991), rat anti-Atg8a primary and appropriate secondary antibodies: Alexa568 conjugated anti-rat (Life Technologies, A11077), Alexa 488 conjugated anti-chicken (Life Technologies, A11039), and Alexa546 conjugated anti-rabbit (Invitrogen, A11035).^3,9,11^ Gel electrophoresis and western blots were done as before, using mouse anti-αTub84B/α-tubulin (DSHB, AA4.3-s), mouse anti-P-S6k/Phospho-RPS6KB1 (Thr389) (Cell Signaling Technology, 9206), rabbit anti-P-Thor/Phospho-EIF4EBP1 (Thr37/46) (Cell Signaling Technology, 2855), rabbit anti-Atg8a, rat anti-Atg13, rat anti-Atg1 and rat anti-Atg9 primary and appropriate alkaline phosphatase-conjugated secondary antibodies: anti-rabbit (Sigma-Aldrich, A3812), anti-rat (Jackson ImmunoResearch, 112-055-003), and anti-mouse (Millipore, AP124A).^3,9,11,33^
