# Supplementary material for: Loss of Drosophila Vps16A enhances autophagosome formation through reduced Tor activity
Source: Autophagy. 2015 Sep 14;11(8):1209–15. doi: 10.1080/15548627.2015.1059559 (PMC4590676; doi:10.1080/15548627.2015.1059559)
Supplement: 1059559_supplemental_files.zip [file kaup-11-08-1059559-s001.zip › 1059559 supplemental files.docx]

**Loss of *Drosophila* Vps16A enhances autophagosome formation through reduced TOR activity**

Szabolcs Takáts, Ágnes Varga, Karolina Pircs, Gábor Juhász

**Supplemental data**

**Supplemental Table and Figure Legends**

**Table S1.** Detailed genotype data. For multiple different genotypes listed on the same panel (such as in the case of panels showing western blots, PCRs or larvae), genotypes are listed as they appear from left to right in the panel.

**Figure S1.** Expression of various proteins in control animals and *Vps16A* and *Syx17* mutants. Loss of *Vps16A* increases the protein level of the selective autophagy cargo ref(2)P/SQSTM1/p62 far beyond that seen in larvae lacking the autophagosomal SNARE Syx17 or in controls. There is no difference in the overall level of core autophagy proteins Atg1 or Atg9 among the three genotypes. Note that no phosphatase inhibitors were used in this experiment to make it possible to estimate the total amount of Atg1, as this protein is also subject to regulation by phosphorylation.

**Figure S2.** Additional HOPS complex phenotypes and interactions with Tor signaling. (**A to B'**) Ultrastructural analysis. Loss of *Vps16A* results in the large-scale accumulation of double-membrane autophagosomes in fat body cells of larvae starved for 4 h in 20% sugar solution, which is suppressed by fat body-specific forced expression of Rheb (**B**), while autophagosomes still accumulate in tracheal epithel cells where Rheb is not overexpressed (**B'**). (**C to F**). Phalloidin staining to help visualizing cell size. Activation of Tor kinase by overexpression of Rheb or knockdown of *gig/Tsc2* in GFP-positive fat cells promotes cell growth relative to neighboring cells in larvae starved for 48 h in water (**C and E**). Expression of Rheb or *gig/Tsc2* RNAi also promotes fat cell growth in chronically starved *Vps16A*-mutant larvae (**D and F**). (**G**) Loss of *CG32350/Vps11* causes a developmental delay, which is partially rescued by low-level expression of Rheb. Note that fully formed adult flies are visible within the pupal case in control animals on day 10 after egg laying, whereas *CG32350/Vps11* mutants are still larvae. Expression of Rheb promotes the development of *CG32350/Vps11* mutants, as they form pupae by day 10. (**H**) Hypomorphic *lt/Vps41*-mutant larvae grow slower than their heterozygous siblings, based on the size difference of larvae on day 4 after egg laying. Scale bars: 1 µm (**A, B, B’**), and 20 µm (**C to F**).
