# Supplementary material for: Loss of Drosophila Vps16A enhances autophagosome formation through reduced Tor activity
Source: Autophagy. 2015 Sep 14;11(8):1209–15. doi: 10.1080/15548627.2015.1059559 (PMC4590676; doi:10.1080/15548627.2015.1059559)
Supplement: 1059559_supplemental_files.zip [file kaup-11-08-1059559-s001.zip › 1059559 Table S1.docx]

| **Panel** | **Genotype** |
| --- | --- |
| Figure 1A | *w[1118]/w[1118]* |
|  | *Vps16A[d32]/Df(3R)BSC507* |
|  | *Syx17[LL06330]/Df(3L)Exel8098* |
| Figure 1B | *w[1118]/w[1118]* |
|  | *Vps16A[d32]/Df(3R)BSC507* |
|  | *Syx17[LL06330]/Df(3L)Exel8098* |
| Figure 1C | *hsFlp/+; QUAS-mCD8-GFP/+; ET49-QF, FRT82B tub-QS/FRT82B Vps16A[d32]* |
| Figure 1D | *UAS-Venus-raptor/+; hs-Gal4/+* |
| Figure 1E | *UAS-Venus-raptor/+; hs-Gal4, Df(3R)BSC507/Vps16A[d32]* |
| Figure 1F | *w[1118]/w[1118]* |
|  | *Vps16A[d32]/Df(3R)BSC507* |
|  | *UAS-Rheb/+; Vps16A[d32]/hs-Gal4,Df(3R)BSC507* |
|  | *Syx17[LL06330]/Df(3L)Exel8098* |
| Figure 2A | *hsFlp, Act>CD2>Gal4, UAS-mCD8-GFP/+; UAS-Rheb/+; Vps16A[d32]/ Df(3R)ED5339* |
| Figure 2B | *Vps16A[d32]/Df(3R)BSC507* |
| Figure 2C | *UAS-Rheb/cg-Gal4; Vps16A[d32]/ Df(3R)ED5339* |
| Figure2D | *w[1118]/w[1118]* |
|  | *Vps16A[d32]/Df(3R)BSC507* |
|  | *Vps16A[d32]/hs-Gal4,Df(3R)BSC507* |
|  | *UAS-Rheb/+; Vps16A[d32]/hs-Gal4, Df(3R)BSC507* |
| Figure 2E | *Vps16A[d32]/Df(3R)BSC507* |
|  | *UAS-Rheb/+; Vps16A[d32]/hs-Gal4, Df(3R)BSC507* |
| Figure S1 | *w[1118]/w[1118]* |
|  | *Vps16A[d32]/Df(3R)BSC507* |
|  | *Syx17[LL06330]/Df(3L)Exel8098* |
| Figure S2A | *Vps16A[d32]/Df(3R)BSC507* |
| Figure S2B | *UAS-Rheb/cg-Gal4; Vps16A[d32]/ Df(3R)ED5339* |
| Figure S2C | *hsFlp, Act>CD2>Gal4, UAS-mCD8-GFP/+; UAS-Rheb/+* |
| Figure S2D | *hsFlp, Act>CD2>Gal4, UAS-mCD8-GFP/+; UAS-Rheb/+; Vps16A[d32]/ Df(3R)ED5339* |
| Figure S2E | *hsFlp, Act>CD2>Gal4, UAS-mCD8-GFP/+; UAS-gig RNAi{KK100646}/+* |
| Figure S2F | *hsFlp, Act>CD2>Gal4, UAS-mCD8-GFP/+; UAS-gig RNAi{KK100646}/+; Vps16A[d32]/ Df(3R)ED5339* |
| Figure S2G | *w[1118]/w[1118]* |
|  | *CG32350[LL06553]/CG32350[LL06553]* |
|  | *hs-Gal4/UAS-Rheb; CG32350[LL06553]/CG32350 [LL06553]* |
| Figure S2H | *w[1118]/w[1118]* |
|  | *lt[11]/Df(2L)lt45* |

**Table S1.** *Drosophila* genotypes used in this study.
